# Review of policies, data, and interventions to improve maternal nutrition in Afghanistan

**DOI:** 10.1111/mcn.13003

**Published:** 2020-04-15

**Authors:** Christine Kim, Ghulam Farooq Mansoor, Pir Mohammad Paya, Mohammad Homayoun Ludin, Mohammad Javed Ahrar, Mohammad Omar Mashal, Catherine S. Todd

**Affiliations:** ^1^ Department of Health Policy and Management, Gillings School of Global Public Health University of North Carolina at Chapel Hill Chapel Hill North Carolina USA; ^2^ FHI 360/Integrated Hygiene, Sanitation, and Nutrition (IHSAN) project Kabul Afghanistan; ^3^ Public Nutrition Directorate, Ministry of Public Health, Islamic Republic of Afghanistan Kabul Afghanistan; ^4^ Rural Water Supply and Irrigation Programme (RuWATSIP) Department Ministry of Rural Rehabilitation and Development (MRRD) Islamic Republic of Afghanistan Kabul Afghanistan; ^5^ Division of Reproductive, Maternal, Newborn, and Child Health FHI 360 Durham North Carolina USA

**Keywords:** Afghanistan, fragile and conflict‐affected, maternal anaemia, maternal nutrition, micronutrients, multisectoral approach, nutrition policy

## Abstract

Malnutrition contributes to direct and indirect causes of maternal mortality, which is particularly high in Afghanistan. Women's nutritional status before, during, and after pregnancy affects their own well‐being and mortality risk and their children's health outcomes. Though maternal nutrition interventions have documented positive impact on select child health outcomes, there are limited data regarding the effects of maternal nutrition interventions on maternal health outcomes globally. This scoping review maps policies, data, and interventions aiming to address poor maternal nutrition outcomes in Afghanistan. We used broad search categories and approaches including database and website searches, hand searches of reference lists from relevant articles, policy and programme document requests, and key informant interviews. Inclusion and exclusion criteria were developed by type of source document, such as studies with measures related to maternal nutrition, relevant policies and strategies, and programmatic research or evaluation by a third party with explicit interventions targeting maternal nutrition. We abstracted documents systematically, summarized content, and synthesized data. We included 20 policies and strategies, 29 data reports, and nine intervention evaluations. The availability of maternal nutrition intervention data and the inclusion of nutrition indicators, such as minimum dietary diversity, have increased substantially since 2013, yet few nutrition evaluations and population surveys include maternal outcomes as primary or even secondary outcomes. There is little evidence on the effectiveness of interventions that target maternal nutrition in Afghanistan. Policies and strategies more recently have shifted towards multisectoral efforts and specifically target nutrition needs of adolescent girls and women of reproductive age. This scoping review presents evidence from more than 10 years of efforts to improve the maternal nutrition status of Afghan women. We recommend a combination of investments in measuring maternal nutrition indicators and improving maternal nutrition knowledge and behaviours.

**Key messages**

- Policies and strategies to improve nutrition more recently have not only shifted towards multisectoral efforts but also specifically targeted adolescent girls and women. There is a dearth of evidence on the effectiveness of interventions targeting maternal nutrition in Afghanistan.
- Ensuring access to quality nutrition care for women and adolescent girls will have intergenerational health and nutrition gains. However, measures of nutrition outcomes and intervention/service coverage of pregnant women, WRA, and adolescent girls are rarely collected and reported.
- Maternal nutritional status is critical to the welfare of the population, and programmes need to invest in systematic implementation research and more robust outcome monitoring and evaluation to understand feasible delivery mechanisms and programme effectiveness.

## 1 INTRODUCTION

From 1990 to 2015, the global maternal mortality ratio (MMR) declined by 44%. However, many women continue to die from preventable and treatable complications, especially in low‐ and middle‐income countries (LMIC), in which the MMR was 436 deaths versus 216 globally per 100,000 live births in 2015 (UNICEF, 2017). Malnutrition and micronutrient deficiencies contribute directly and indirectly to maternal morbidity and mortality. Evidence indicates that maternal malnutrition, measured through mid‐upper arm circumference (MUAC), is strongly associated with the risk of maternal mortality (Christian et al., 2008; Sikder et al., 2014). Anaemia is a risk factor for maternal mortality and morbidity due to exacerbated effects of haemorrhage and impaired clotting capacity, and calcium deficiency increases the risk of hypertensive disorders of pregnancy, principally eclampsia (Black et al., 2008; Daru et al., 2018; Maternal and Child Nutrition Study Group, 2013; Rush, 2000). Christian et al., 2000 also found that night blindness, a symptom of vitamin A deficiency, was predictive of a 3.8 times greater risk of maternal death in Nepal. A recent nested study compared reported maternal complications among postpartum women in intervention and control areas for a cluster‐randomized trial of intensified maternal nutrition counselling and micronutrient supplementation added to an existing home‐based antenatal/postnatal care (ANC/PNC) programme in Bangladesh (Todd et al., 2019). Odds of reported retained placenta, postpartum bleeding, and postpartum infection or fever were significantly higher among women living in the control areas. Anaemia, micronutrient deficiencies, and attendant pregnancy complications also contribute to delayed maternal recovery from delivering a child, low birth weight, and neonatal morbidity, which in turn contribute to intergenerational malnutrition from mothers to their babies (Christian, Mullany, Hurley, Katz, & Black, 2015).

The 2013 Lancet Maternal and Child Nutrition Series reemphasized global commitments to focus on the first 1,000 days of life for improving nutrition outcomes among children (Black et al., 2013). Maternal nutrition—defined as the nutrition needs of women during antenatal and postnatal periods and sometimes also to the period prior to conception, even as early as adolescence in contexts where early childbearing is normative (The Manoff Group, 2012)—affects the birth outcomes, growth, and development of their children as well as mothers' own well‐being and mortality risk (Black et al., 2013; Goudet, Murira, Torlesse, Hatchard, & Busch‐Hallen, 2018).

Despite associations between adequate maternal nutrition and positive maternal and child health outcomes, few studies have documented the impact of nutrition interventions focused on maternal preconception and pregnancy. Limited high‐quality evidence exists for multisectoral nutrition interventions, particularly on the incorporation of nutrition‐sensitive approaches such as water, sanitation, and hygiene (WASH); agriculture; and food security into existing nutrition‐specific programming (Maternal and Child Nutrition Study Group, 2013). Additionally, most maternal nutrition interventions measure newborn and child health indicators as primary outcomes with relatively fewer assessing maternal health indicators (Christian et al., 2015). A systematic review of evidence‐based nutrition programmes taken to scale found that the following components improve birth outcomes and overall household nutrition: micronutrient supplementation, food fortification and supplements, nutrition education and counselling, and conditional cash transfers (Victora et al., 2012). The extent to which these interventions reduce maternal complications is less rigorously documented. Goudet et al. (2018) identified nine studies in their systematic review measuring the effectiveness of the approaches to improve the coverage of maternal nutrition interventions in South Asia. They found that a range of individual and health service delivery barriers, such as education and access to services, affects coverage by nutrition interventions. The detected range of interventions (e.g., iron and folic acid [IFA] and calcium supplementation and maternal nutrition counselling) and evidence regarding their efficacy were both limited. Multisector nutrition studies were not included, and no studies from Afghanistan were deemed eligible for inclusion. Recent studies on WASH and nutrition have shown limited effects on reducing child malnutrition outcomes, indicating that WASH is necessary but not sufficient, because malnutrition has various underlying causes (Coffey & Spears, 2018; Humphrey et al., 2018). Thus, we believe a multisectoral approach is needed to address malnutrition.

The prevalence of malnutrition among the general population, especially women and children, is significantly higher among fragile and conflict‐affected states, because factors such as forced migration, food insecurity, and restricted access to humanitarian aid can affect nutrition status and exacerbate poor health outcomes (Kinyoki et al., 2017; Seal, 2018; Taylor, Perez‐Ferrer, Griffiths, & Brunner, 2014). Similarly, maternal morbidity and mortality are highest in conflict‐affected countries (Urdal & Che, 2013). Afghanistan, in South Asia, is among the most fragile countries (Fund for Peace, 2018; World Bank, 2019) and has the highest MMR in South Asia (638 deaths/100,000 live births; Kassebaum et al., 2016; UNICEF, 2017). In Afghanistan, effectively addressing maternal malnutrition could result in health sector gains by reducing maternal morbidity and mortality and child stunting and by improving overall survival, growth, and development of children. Understanding the available maternal nutrition data in Afghanistan may improve knowledge sharing, evidence generation, policy development, and programme implementation in the country and other fragile contexts facing similar challenges.

This scoping review maps policies, data, and interventions aiming to address maternal anaemia and poor maternal nutrition outcomes in Afghanistan and to determine the extent of multisectoral involvement in these efforts. We build on a previous multisector nutrition review that was conducted in 2010 and systematically focused on maternal nutrition (Levitt, Kostermans, Laviolette, & Mbuya, 2010). Our objectives were to (1) identify relevant maternal nutrition policy and programmes and describe the degree to which multisectoral approaches were engaged; (2) describe main findings across policy, strategy, programming, and monitoring within available peer‐reviewed evidence and grey literature on maternal nutrition in Afghanistan; and (3) critically appraise data summarizing maternal nutrition status and relevant programme coverage, knowledge, and gaps in Afghanistan.

## 2 MATERIALS AND METHODS

### 2.1 Study design

Overall, we followed the scoping review framework proposed by Arksey and O'Malley (Arksey & O'Malley, 2005) and enhanced by Levac, Colquhoun, and O'Brien (2010). A scoping exercise was deemed appropriate rather than a systematic review to map the range of published and grey literature, policy, and data sources on maternal nutrition in Afghanistan and to better define gaps within existing practice and research. We applied a two‐step process for identifying and selecting sources and engaging stakeholders (Levac et al., 2010). The first step comprised a rapid desk review (conducted in August 2017) along with stakeholder interviews to identify available sources and perceived information gaps within both child stunting and maternal nutrition (IHSAN project/FHI 360, MoPH [Afghanistan], MRRD [Afghanistan], & USAID, 2018). The second step was a follow‐up systematic literature search and a second hand‐search of relevant maternal nutrition content conducted between September 2018 and February 2019.

### 2.2 Search strategy

The rapid desk review conducted a literature search for child and maternal nutrition documents; details are reported elsewhere (IHSAN project/FHI 360 et al., 2018). The follow‐up systematic literature search was conducted across 13 databases: MEDLINE/PubMed, Popline, Embase, Global Health, Academic Search Premier, CINAHL Plus with Full Text, EconLit, Education Full Text (H.W. Wilson), Environment Complete, ERIC, GreenFILE, Middle Eastern & Central Asian Studies, and Web of Science. Example search terms included (anaemia OR “micronutrient deficiency” OR “iron deficiency” OR anaemia, iron‐deficiency[Mesh] OR “dietary diversity” OR “MUAC” OR “middle upper arm circumference” OR “nutritional status” OR “pregnancy complications” OR “pelvic contracture” OR “knowledge” OR “behaviour change) AND (pregnancy OR pregnant OR pregnant women[Mesh] OR maternal OR “women of reproductive age” OR fertility) AND Afghanistan[Mesh]). Reference lists from 14 identified review papers were hand‐searched for additional sources. A second purposive hand‐search was conducted for relevant grey sources from websites of government ministries, nongovernmental organization (NGO) implementing partners in the health sector, and donors.

### 2.3 Source selection

Before selecting sources, we agreed on broad inclusion criteria applied at the first search stage to maximize the initial range of documents. Documents included in the review had or addressed a maternal nutrition measure, policy, or strategy in Afghanistan. We then refined inclusion criteria by discussing source selection based on the first phase of abstract review, which delineated available and relevant maternal nutrition content and sources (Levac et al., 2010). This approach helped alleviate uncertainties surrounding source selection, given the broad research question and goal of including grey literature sources.

To map the current maternal nutrition situation in Afghanistan, we categorized our sources into peer‐reviewed articles, grey literature reports, data sources, and policy documents. Box 1 displays the final eligibility criteria.

**BOX 1:** Eligibility criteria

Inclusion criteria			
Peer‐reviewed article	Grey literature	Data source	Policy document
Peer‐reviewed research on nutrition, food security, and/or WASH interventions Measures at least 1 maternal nutrition health outcome or nutrition‐related knowledge or behaviour (includes hygiene) Specific to Afghanistan	Programmatic research or evaluation done by third party Explicit interventions/programmes on nutrition, food security, and/or WASH with expected changes in maternal nutrition status Implemented in Afghanistan	Measurement of maternal nutrition‐related knowledge, behaviours, or outcomes Data collected from Afghanistan	All maternal nutrition‐relevant government policies/strategies included
**Exclusion criteria**			
Not publicly disseminated or unavailable to public upon request from source Not specific to Afghanistan Target population of Afghan refugees no longer in the country or not including women of childbearing age or adolescent girls Not written in English Global or multicountry study without specific data from Afghanistan, rather only aggregated global or regional estimates Agriculture, food security, economic development, or water and sanitation‐related programming and/or research without explicit maternal nutrition‐related component or measures			

We included all relevant data sources and policy documents for analysis. Only published articles and grey literature reports underwent full‐text screening; we excluded brief reports, project promotional pamphlets, and other documents without a primary data source. Two independent reviewers screened all abstracts/titles (C. K. and C. S. T.) and all full texts (G. F. M. and M. O. M.). Any disagreements on source inclusion were resolved by the two reviewers, and when necessary, by a third reviewer. We did not appraise source quality because we aimed to include all relevant content to provide the full landscape of maternal nutrition programming.

### 2.4 Citation management and data extraction

All citations were imported into Endnote X7 (Clarivate Analytics, Philadelphia). Duplicate citations were removed manually during screening. Data were extracted into a spreadsheet, where descriptive information was extracted based on specified categories.

### 2.5 Analysis

We first collated and summarized the scope of available sources. We then described any evaluated nutrition programmes with at least one maternal nutrition component and appraised the extent to which multisectoral approaches were used. We synthesized information identifying changes to priority programme areas and outcomes over time in data sources and policy documents, respectively, with iterative identification of gaps.

## 3 RESULTS

### 3.1 Scope of sources

**FIGURE 1:**
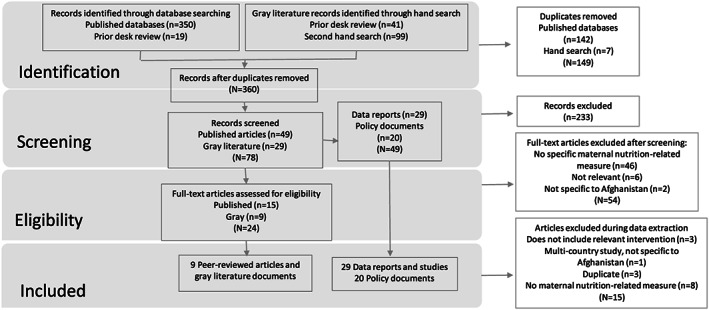
Flow chart

After removing duplicates from combined search results, we screened 360 documents by abstract and title. All relevant data reports and policy documents were included. Full‐text screening was conducted for 15 peer‐reviewed articles and nine grey literature documents. In summary, we found 29 data reports, 20 policy documents, and nine full‐text studies (peer‐reviewed articles and grey literature programme reports) relevant to maternal nutrition in Afghanistan that met the inclusion criteria for this scoping review (Figure 1).

In the following section, we describe how maternal nutrition status and related indicators have been measured in Afghanistan. We then provide an overview of the government of Afghanistan's policy response to maternal nutrition. Finally, we present information from eligible full‐text documents about maternal nutrition‐specific and nutrition‐sensitive interventions that aimed to improve the nutrition status of adolescent girls, women of reproductive age (WRA), and pregnant women in Afghanistan.

### 3.2 Assessing the nutrition situation among adolescent girls and pregnant women: Data sources

#### 3.2.1 Overview of data sources

Since 2002, sources reporting maternal nutrition‐related indicators in Afghanistan have increased in number and quality (Table 1). Before the 2004 National Nutrition Survey (NNS), six data sources were generated, half of which were Multiple Indicator Cluster Surveys (MICS), in 1997, 2000, and 2003. A household food security study was also conducted in all regions except for eastern Afghanistan, and a focused study was conducted on vitamin A deficiency in Kabul city (Lautze, 2002; Mihora et al., 2004). Eight data sources were generated from 2005 to 2012, including National Risk and Vulnerability Assessments (NRVAs) from 2005, 2007, and 2011; MICS in 2011; three primary subnational studies; and one secondary data analysis. The NRVA (now the Afghanistan Living Conditions Survey [ALCS]; CSO [Afghanistan], 2016) was recently conducted in 2016–2017 and provides multisectoral estimates on poverty, food security, health, agriculture, and other indicators. The NRVA/ALCS is implemented routinely at two‐ to three‐year intervals. Other than national surveys, data sources include an iodine deficiency case‐control study among households in an NGO programme area (Oberlin, Plantin‐Carrenard, Rigal, & Wilkinson, 2006), formative research on food systems for comprehensive anaemia control in northern Afghanistan (Levitt, Stoltzfus, Pelletier, & Pell, 2009), an ANC utilization study in Balkh Province (Hadi, Mujaddidi, Rahman, & Ahmed, 2007), and a secondary analysis of pilot national nutrition surveillance data (Johnecheck & Holland, 2005).

**TABLE 1:** Summary of data sources on maternal nutrition

		2004 NNS and earlier	2005–2012	2013 NNS and later
Number of data sources		6	8	15
Type of data collection	Primary data collection reports	6	7	12
	Secondary data analysis	0	1	4
	Mixed use of data	0	0	1
Geographic representation	National	1	1	3
	National and/or regional	4	3	1
	National and/or provincial	0	2	5
	Specific area to programme/facility and/or not representative of any region	1	2	8
Implementers	NGO	2	2	4
	Government/CSO	1	4	4
	UN or World Bank	0	1	3
	Academic institution/research organization (national or Int'l)	3	1	5
Maternal nutrition‐related measures	Maternal nutrition status			
	Underweight (BMI < 18.5)	1	1	2
	Overweight (BMI 25–29.9), obese (BMI ≥30)	1	1	2
	MUAC scores for global acute malnutrition, severe acute malnutrition, and moderate acute malnutrition	0	0	1
	Vitamin A deficiency/night blindness	2	0	2
	Visible goitre/iodine deficiency	1	1	1
	Anaemia/Iron deficiency/	1	1	4
	Zinc deficiency	0	0	1
	Vitamin D deficiency	0	0	1
	Coverage of maternal health and nutrition services			
	ANC use and composition	0	1^a^	1^b^
	Vitamin A supplementation	2	0	2
	Household iodized salts	2	2	1
	IFA supplementation	0	0	3
	Household WASH			
	Safe drinking water	3	5	5
	Household water insecurity	1	0	0
	Improved sanitation	2	5	6
	Handwashing with soap/ash, (@key times)	1	2	4
	Household food insecurity			
	Sufficient food last week	1	0	0
	Household perception of food security	0	2	0
	Dietary diversity	0	1	4
	Calorie deficiency	0	1	0
	Protein deficiency	0	1	1
	Hunger scale	0	0	1
	Food insecure population	0	0	2
	Households receiving food aid	1	0	0
	Households owning garden plot	0	0	2
	Acceptable food consumption/diet, coping mechanisms	1	0	2
Sources without nutrition indicators	Qualitative study	0	0	3
	Statistical analyses on variables associated with nutrition outcomes	0	0	3^c^

a Weight measurement, urine test, blood test, iron tablet/syrup.

b Nutrition counselling and information.

c One source on anaemia and odds of taking IFA tablets, one on BMI and associated risk factors, and one on the effect of floods on probability of anaemia.

After the 2013 NNS, 15 data sources provided maternal nutrition information, encompassing a wider variety compared with previous periods. The Afghanistan Health Survey (AHS; The Royal Tropical Institue and Silk Route Training and Research Organization, 2016) and Demographic and Health Survey (DHS; CSO, MoPH [Afghanistan], The DHS Program,, & ICF International, 2016) are both national health sector‐specific cross‐sectional household surveys. The AHS provides national and provincial level estimates on priority health sector indicators used to evaluate health service delivery projects. The Afghanistan DHS provides similar estimates on a range of demographic and health indicators. The Standardized Monitoring and Assessment of Relief and Transitions (SMART) survey is a programme‐related rapid nutrition assessment aiming to provide rapid coverage estimates and programme area information on population nutrition status and WASH behaviours (ACF International, 2014; Habib, 2017). Finally, the national nutrition surveillance bulletin provides quarterly surveillance information across 175 facility and 953 community sentinel sites (MoPH [Afghanistan], 2017).

We organized maternal nutrition‐related measures into five categories: maternal nutrition status, micronutrients, food security, use of maternal health services, and WASH.

#### 3.2.2 Maternal nutrition status

**FIGURE 2:**
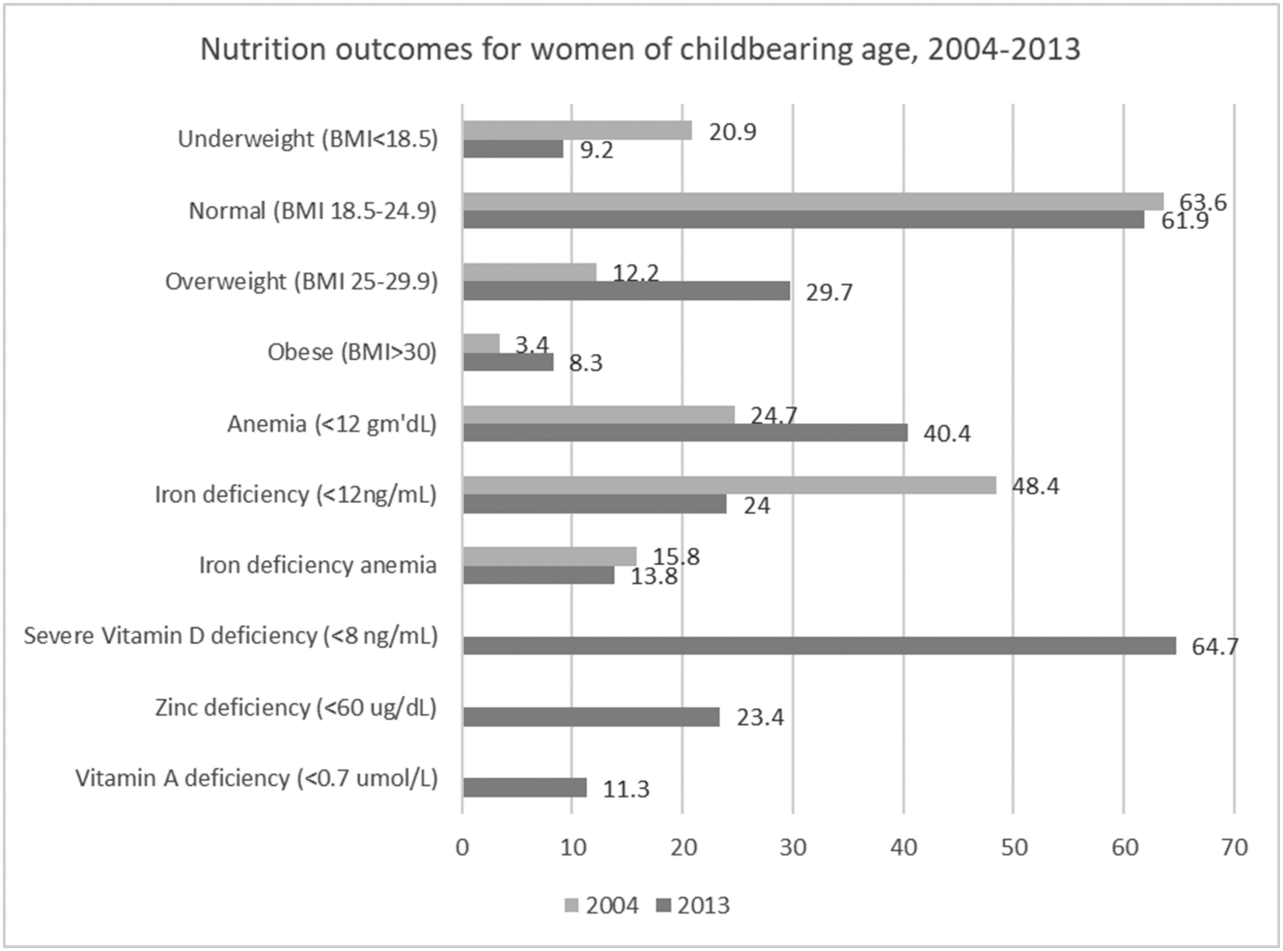
Nutrition outcomes for women of reproductive age, NNS 2004–2013

Figure 2 displays key nutrition indicators from 2004 and 2013 among WRA. Nutrition outcomes measured among non‐pregnant WRA are based on body mass index (BMI in kg/m^2^) to determine underweight (BMI < 18.5), normal (BMI 18.5–24.9), overweight (BMI 25–29.9), and obese (BMI > 30) status. Before the 2013 NNS, underweight and overweight/obese BMI data were reported only by the 2004 NNS and by Oberlin et al. (2006; MoPH [Afghanistan], 2004).

Between 2004 and 2013, although the proportion of underweight women decreased (20.9% to 9.2%), there was an increase of overweight (12.2% to 29.7%) and obese (3.4% to 8.3%) women (MoPH [Afghanistan], 2004; MoPH [Afghanistan] & UNICEF, 2013). Further, there were significant geographic variations in underweight, overweight, and obese prevalence among WRA, as well as disparities by wealth in the dual burdens of underweight and overweight/obese in districts of the north, northeast, central, and central highland regions (Akseer et al., 2018). The prevalence of obesity among women overall and in an eastern city, Jalalabad, was 27.4% and 35.9%, respectively (Saeed, 2015). Among 55 peri‐urban women, 20% were underweight (Oberlin et al., 2006). In a secondary analysis of 2013 NNS data, maternal BMI was associated with many factors, including household wealth status, education level, region, food insecurity, unimproved water and sanitation facilities, age, ethnolinguistic status, and parity (Akseer et al., 2018). Low maternal height was not reported by any of the data sources. This presents a knowledge gap considering the associations found between low birth weight and poor linear growth in children born to women with low maternal height (Addo et al., 2013; Britto et al., 2013). In Afghanistan, the NNS measured height as part of BMI calculations, but in its analysis and in secondary analyses of NNS data, there are no mentions of data presented on low (<145 cm) maternal height. Further, nutrition status of WRA based on MUAC scores was only done in small area SMART surveys (Habib, 2017).

#### 3.2.3 Micronutrient deficiencies

**FIGURE 3:**
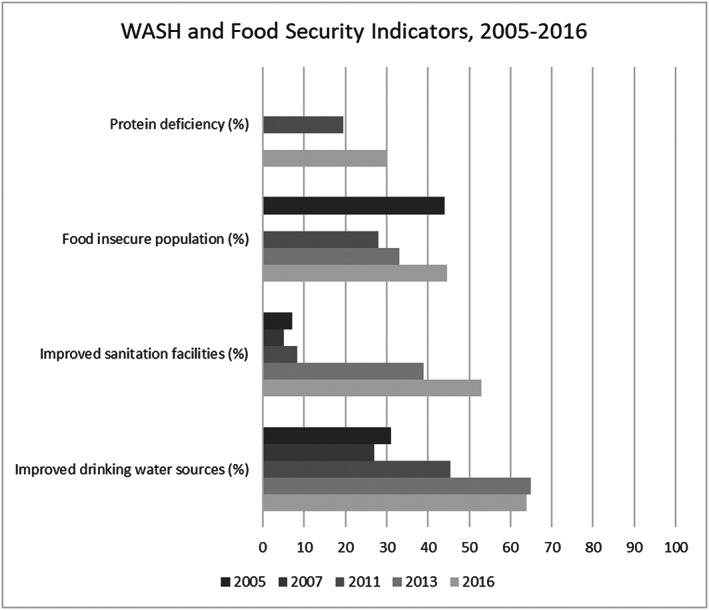
WASH and food insecurity indicators from the NRVA/ALCS, 2005–2016

The 2013 NNS captured widespread micronutrient deficiencies among WRA: 40.4% were anaemic, 23.4% had zinc deficiency, 11.3% had vitamin A deficiency, and 64.7% had severe vitamin D deficiency (MoPH [Afghanistan] & UNICEF, 2013). The most commonly reported micronutrient deficiencies and their corresponding supplementation interventions are vitamin A and anaemia/iron deficiency. Zinc and vitamin D deficiencies were not studied among WRA before 2013. Mihora et al. (2004) found that vitamin A deficiency was a moderate to severe health problem among lactating mothers in Kabul city, yet postpartum vitamin A supplementation was almost nonexistent during the 2004 study. In 2006, Oberlin et al. (2006) studied the presence of goitre among WRA in an area slightly north of Kabul; of 62 women, 30 had goitre. Studies assessing associations between anaemia and other diseases or natural disasters were also conducted after 2013. Howard, Enayatullah, Mohammad, Mayan, and Shamszai (2015) found no association between high prevalence of anaemia among pregnant women and malaria in Nangarhar Province. Oskorouchi, Nie, and Sousa‐poza (2018) found that floods were significantly associated with anaemia through two possible routes: (1) increased waterborne diseases with attendant diarrhoea and malabsorption and (2) decreased vitamin A‐rich food intake, which decreased serum retinol. Figure 3 displays micronutrient deficiency trends from 2004 to 2013 among WRA. Although iodine deficiencies among WRA have been reported, the most commonly used indicator has been the prevalence of urinary iodine concentration < 100 μg/L, rather than the recommended median value specifically for pregnant and lactating women, representing a notable gap (World Health Organization [WHO], 2013).

#### 3.2.4 Food security

Figure 3 also shows the population‐based increase in protein deficiency and food insecurity between 2005 and 2016. By 2016, 44.6% of the population was food insecure and 30.1% had protein deficiency (CSO, 2018). Levitt et al. (2009) conducted formative research in northern Afghanistan to understand iron‐rich food access and consumption by populations living in irrigated and rain‐fed zones. In both zones, summer and winter diets contained some iron‐rich foods, although differences were found between communities in the summer, such as meat consumption no more than once a week in the rain‐fed zone compared with twice a week in the irrigated zone, with the increased consumption tied to larger social occasions as a means of honouring guests. Households in the irrigated zone owned more food animals and livestock holdings than households in the rain‐fed zone. In a shared plate culture, where men and women in a household typically eat together unless entertaining guests, it is unclear to what extent women consume meat even when it is available. Although women reported having three main meals, the default was bread and tea, especially for breakfast. In a qualitative study, Newbrander, Natiq, Shahim, Hamid, and Skena (2014) found that women reported postpartum diet restrictions due to “cold” (e.g., beef and watermelon) and “hot” (e.g.. beans) food characteristics, which were alleged to cause body pains or were perceived as difficult to digest, respectively, and thus were avoided in the postpartum period, limiting dietary diversity. Although dietary diversity is a proxy indicator for food insecurity, this study found limited dietary diversity was based on social norms rather than actual inaccessibility of food, the latter of which was not directly explored (Hoddinott & Yohannes, 2002).

#### 3.2.5 Use of maternal health and nutrition services

In 2013, among 48.1% of women reporting using ANC services during their most recent pregnancy, less than half received information and counselling about nutritious food in appropriate quantities (MoPH [Afghanistan] & UNICEF, 2013). The 2015 DHS found that 42.4% of women received IFA tablets during pregnancy and 23.2% of postpartum women reported receiving vitamin A supplementation (MoPH [Afghanistan] & CSO [Afghanistan], 2017). Hadi et al. (2007) assessed the specific ANC components pregnant women received. Among the nutrition‐related components, the study found that women who experienced greater difficulty accessing ANC received fewer nutrition‐related services. For example, 20.1% of women who experienced difficulty accessing ANC were weighed compared with 41.1% with easier ANC access; 4.1% vs. 27% received a urine test; and 23.1% vs. 44.5% received iron supplements, respectively. These data reflect three nutrition‐related services provided within ANC, being weighed, receiving counselling about specific dietary needs during pregnancy and lactation, and receiving micronutrient supplementation. There was no measure of whether women were counselled on dietary micronutrient intake or supplementation to prevent maternal complications, management of nausea and food aversion during pregnancy, or appropriate weight gain in pregnancy. These gaps should also be assessed with regard to provider knowledge and awareness.

#### 3.2.6 WASH

WASH strategies and studies considered women's WASH behaviours related to their child's health but did not measure illness and micronutrient deficiencies among women secondary to poor sanitation and hygiene. Figure 3 shows household WASH indicators from the NRVA/ALCS survey rounds from 2005 to 2016.

**FIGURE 4:**
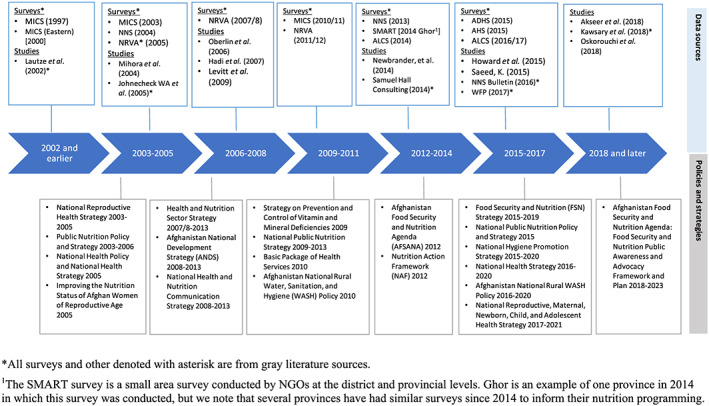
Timeline of data sources and policies relevant to maternal nutrition

### 3.3 Policy responses to maternal malnutrition

A timeline of policy documents developed since 2003 to improve nutrition outcomes in Afghanistan is presented in Figure 4 alongside the generation of nutrition‐related data. The Public Nutrition Policy 2003 and National Health Policy 2005 focus on child malnutrition and household access to fortified foods and micronutrient intake but do not explicitly outline maternal malnutrition strategies (MoPH [Afghanistan], 2005; Public Nutrition Department (PND MoPH) [Afghanistan], 2003). Policies aiming to improve maternal nutrition status initially focused on the health sector. The 2003 National Reproductive Health Strategy integrates nutrition strategies to improve reproductive health through the safe motherhood initiative, specifically during ANC, PNC, and family planning counselling. Maternal nutrition strategies focus on micronutrient supplementation (IFA, calcium, and vitamin A) and nutrition education at community and facility levels (Reproductive Health Taskforce (Women's and Reproductive Health Directorate) [Afghanistan MoPH], 2005). A prepolicy document providing recommendations for generating maternal nutrition strategy was collaboratively developed by the Public Nutrition Department and UNICEF in 2005 (PND MoPH [Afghanistan], 2005). A stakeholder meeting was convened in 2005 where strategy areas were prioritized, but, due to supporting project closure and BPHS revision, a maternal nutrition strategy and policy were not fully developed. Although no maternal nutrition‐specific strategy has been developed, many recommended approaches are included in subsequent health sector policies and strategies for improving maternal nutrition. These approaches include IFA supplementation during adolescence, pregnancy, and lactation; multimicronutrient supplementation especially for pregnant and lactating women, and adolescent girls; and household food‐based approaches (flour and salt fortification and dietary diversification; PND MoPH) [Afghanistan], 2010). Half of the recommended approaches in the policies and strategies targeted micronutrient supplementation, for which the MoPH developed a separate strategy on prevention and control of vitamin and mineral deficiencies in 2009. Another separate MoPH strategy on IYCF was developed in 2009. WHO introduced updated recommendations for ANC in 2016, which included nutrition interventions. At least eight ANC visits are now recommended and multimicronutrient supplementation is not recommended; however, policies in the Afghan health sector have yet to align with these new global recommendations and IFA is the only micronutrient supplement widely available in public sector facilities (World Health Organization, 2016).

Maternal nutrition‐related education and food‐based approaches were incorporated across sectors, particularly messaging promoting healthy foods for pregnant women, increased caloric intake during pregnancy, and overall household food safety and hygiene (Department Ministry of Rural Rehabilitation and Development (MRRD) Afghanistan, 2010; Government of the Islamic Republic of Afghanistan, 2012; MoPH [Afghanistan], 2008). The 2008 Afghanistan National Development Strategy (ANDS) was the country's first extensive multisector poverty reduction document, including malnutrition but not specific to maternal nutrition. In 2012, the Nutrition Action Framework (NAF) and the Afghanistan Food Security and Nutrition Agenda (AFSANA, changed to AFSeN in April 2017) were developed, and these prioritize multisector action to improve nutrition (AFSeN‐Technical Secretariat, 2018; Islamic Republic of Afghanistan, 2012). Although these policies highlight the intergenerational effects of malnutrition and importance of maternal diet and nutrition, they do not include maternal nutrition‐related targets. By 2015, follow‐up strategies within the Rural WASH Strategy, National Health Strategy, and the National Reproductive, Maternal Newborn, Child, and Adolescent Health Strategy continued to include objectives expanding access to nutrition‐specific and nutrition‐sensitive interventions. Compared with previous policy and strategy documents, there was greater inclusion of nutrition and micronutrient needs of adolescent girls and women. For example, strategies include targeting adolescent girls in schools and at health facilities to provide micronutrient supplements. Although these policies provide a comprehensive review of the nutrition situation of girls and women at preconception, pregnancy, and during their child's first 1,000 days of life, they do not include targets to measure progress on maternal nutrition outcomes or approaches to sociocultural barriers best addressed through nutrition‐sensitive interventions. One example of such a policy gap is a collaborative policy to retain young women in late primary and secondary school to both reduce risk of early marriage and pregnancy and to institute school‐based nutrition‐specific interventions, such as IFA supplementation. Maternal nutrition policies were not generated before the most recent 2016 Nutrition Strategy, in which it is made explicit by the Public Nutrition Department. Although few maternal nutrition strategies were mainstreamed through policies for reproductive health, the capacity to properly implement maternal nutrition interventions was insufficient in other departments. Table 2 summarizes relevant policies and strategies.

**TABLE 2:** Summary of policies and strategies on maternal nutrition in Afghanistan

Year	2003–2005	2006–2008	2009–2011	2012–2014	2015–2017	2018
Government agency (*n*)	MoPH (4)	MoPH (2)Government (1)	MoPH (3)MRRD (1)	Government (2)	MAIL (1)MoPH (4)MRRD (1)	Government (1)
Multisectoral considerations	The public nutrition policy and health sector policy recognize multi‐causal nature of malnutrition and need for MoPH collaboration with other ministries. Food‐based approaches included as a recommended strategy for a maternal nutrition strategy.	Health and nutrition sector strategy and nutrition communication strategy acknowledge need for broad‐based interventions to tackle malnutrition, specifically in regards to nutritious foods education/awareness. ANDS is multisector by design and guides the overall development strategy.	Public nutrition strategy and strategy on prevention and control of vitamin and mineral deficiencies acknowledge need for broad‐based interventions to tackle malnutrition, specifically in regard to nutritious foods and education/awareness. MoPH BPHS guidelines are health service delivery specific. WASH policy addresses multisectoral approaches for improving nutrition outcomes and was developed under MRRD with support from other ministries.	AFSANA and NAF address multisectoral approaches for improving nutrition outcomes with WASH and food security and were developed across multiple ministries.	Public nutrition policy and FSN have extensive linkages to food security and food safety strategies and their effect on nutrition. Hygiene promotion strategy covers messages on sanitation, personal hygiene, and food hygiene. Updated WASH strategy. RMNCAH strategy includes nutrition education for adolescents in schools, including micronutrient supplementation (vitamin A, IFA).	MoPH National Health Strategy has a strong health sector focus. AFSeN was developed with multiple ministries and includes both nutrition and food security.
Main maternal nutrition‐related objectives	Household access to fortified foods (e.g., iodized salt) and other micronutrient deficiencies (e.g., scurvy) as well as knowledge and awareness. Maternal nutrition objectives only included in the Reproductive Health policy and prematernal nutrition strategy document.	Increasing the coverage and quality of services to prevent and treat malnutrition among children and adults, government‐wide efforts to recognize nutrition as foundational to development, establish nutrition targets responsible by all sectors, and identify feasible actions to achieve nutrition targets	Increasing the coverage and quality of services to prevent and treat malnutrition among children and adults; reduce the prevalence of major micronutrient deficiency disorders; increase knowledge, awareness, skills, and capacity in public nutrition; deliver nutrition component of BPHS/EPHS;improve access to safe drinking water, increase hygiene awareness and practices	Improve the availability, access, utilization of healthy foods; ensure healthy diets	Largely a continuation of earlier objectives.	Greater political and social commitment to improve the food security and nutrition situation in Afghanistan. Increase financial resources for food security and nutrition Advocate for involvement of private and public sectors and communities in food security and nutrition activities
Key maternal nutrition‐related strategies	Micronutrient fortification; nutrition education, communication, and advocacy; postpartum vitamin A supplementation; birth spacing	In addition to previous strategies, collaborate with other line ministries to address environmental health consequences of poor water supplies and lack of adequate sanitation facilities; recognizing the role, responsibility and potential capacity of the food industry and local Markets in Afghanistan	In addition to previous strategies, adopt a public nutrition approach involving multisectoral interventions; implement strategies through the BPHS and link with food security and other social development programmes; focus on quality salt iodization, flour fortification; hygiene education in schools, community groups, women's groups (particularly on sanitary requirements for young girls); counselling on hygiene and sanitation through augmented community health service provision.	In addition to previous strategies, increasing food availability for food insecure families through food production and dietary diversification, food storage and preservation, and market availability; improving food access for food insecure families (food and cash transfers, food for work, food for assets), community‐based income generation	In addition to previous strategies, take a multipronged approach to address micronutrient deficiency problems, with a special focus on anaemia and iron deficiency anaemia among women of reproductive age. Inclusion of adolescent health through school health services and premarital counselling to prevent early pregnancy in health facilities.	Advocacy to prioritized audiences through meetings, workshops, and seminars, along with a package of nutrition advocacy materials targeted to each audience to build a critical mass of food security and nutrition advocates and promote a national coordinated effort to improve food security and nutrition.
Changes over time in maternal nutrition considerations	Baseline policies and strategies.	Language more specific to environmental factors and linkages beyond health sector, nutrition IEC, service provision, and training; nutrition indicators not part of M&E plan for national policy/strategy.	Identified target groups as women, adolescent girls, and children; expanded target micronutrient deficiencies; mapping of nutrition indicators by source.	Emphasis on adolescent girls' nutritional and hygiene needs, and nutritious food programming.	Identified target groups as women, adolescent girls, and children; expanded target micronutrient deficiencies; mapping of nutrition indicators by source.	Prioritization of advocacy audiences of multisectoral government ministries and authorities; private sector (food producers, importers and retailers); religious leaders; development partners, donors and civil society organizations; and media.
Programming	Salt iodization; antenatal care services including IFA supplementation and tetanus toxoid vaccination; kitchen gardens; health and nutrition education; flour fortification.	Implemented EPHS with BPHS; MoPH Food and Drug Quality Control Department established; MAIL Quality Control Department developed legislation, regulatory frameworks, standards, etc., on certification systems and laboratory testing for food quality and safety; nutrition cluster activated.	Passage of maternity protection act; refurbished MoPH equipment and labs to conduct analyses of water quality, iodized salt, fortified flour, and other food products; Afghan National Standards Authority established as official authority to issue standards and certificates.	Established food and nutrition secretariat and high‐level steering committee; efforts to implement nutrition‐sensitive programmes are increasing as the capacity of the Home Economic Directorate in MAIL is improved and agriculture projects are designed to be more nutrition sensitive.	Promotion of home based food processing, storage and conservation; IEC on food and nutrition issues; food safety standards and control; expansion of nutrition sensitive products (vegetables, fruit) in home gardens and on agricultural land; family health houses with a community midwife; FHAGs support CHWs at health posts; community‐led total sanitation (CLTS)	

Abbreviations: AFSANA/AFSeN, Afghanistan Food Security and Nutrition Agenda; CLTS, community‐led total sanitation; FHAG, Family Health Action Groups; MAIL, Ministry of Agriculture, Irrigation, and Labor; MRRD, Ministry of Rural Rehabilitation and Development; MoPH, Ministry of Public Health; NAF, Nutrition and Agriculture Framework; ODF, Open Defecation Free; RMNCAH, Reproductive, Maternal, Newborn, Child, and Adolescent Health; WASH, water, sanitation, and hygiene.

### 3.4 Nutrition programme responses and multisectoral approaches

Table 3 describes nine intervention studies matching our inclusion criteria. Only one study was conducted before 2013. Target groups varied; five studies focused on pregnant women/WRA and children under 5 years, two on households, one on health facilities, and one on adolescent girls (ages 15–25 years). Here, we present interventions as either those delivered specifically through the health system or those delivered through multisector platforms.

**TABLE 3:** Description of included intervention studies

Source, type, geographic coverage, study type	Target group(s)	Intervention description	Intervention category	Multisectoral approach	Nutrition‐related outcome categories and results	Lessons learned
Grunewald, et al. (2008), rey literature, hand search, subnational, qualitative/descriptive evaluation	Households	20 FAO‐executed projects. Support to household food security, nutrition, and livelihoods, with focus on (1) piloting interventions and (2) building MAIL capacity	Package of interventions	Agriculture and livelihoods interventions with nutrition focus	Developed nutrition education booklets and posters with 9 key messages shared with students who reported putting them on walls for their family; developed guide on improved feeding practices and recipes for afghan children and mothers; poultry, dairy, seed, and integrated livelihoods projects; support to MAIL to promote food security and nutrition; school gardens varied from 160 to 800 m^2^ and reported influencing home gardening and food consumption of teachers' and students' families; 500 women were trained in food processing and kitchen gardens; 14 women trained in produce and poultry marketing.	Knowledge increase not quantitatively measured (implementation by NGOs and not measured but reported increases in monitoring reports); range of interventions left little time to test and accumulate sufficient experiences; questions remain on right target group, no thorough analysis of different models used, suitability of messages, calculations of costs/benefits not done, more analysis is needed; low rates of land ownership/access to resources for women to implement project components due to insufficient ownerships of resources; practical livelihood support to increase production, diversify food production, food processing and conservation, and marketing should be provided with nutrition education.
The World Bank (2013), grey literature, national, qualitative assessment	Pregnant women	BPHS IFA supplementation for pregnant women	Micronutrients	None	Discrepancies found between the types and doses of IFA supplements listed in the BPHS essential drugs list; anaemia cut‐off ranges not adjusted for higher altitude; conflicting policy guidance between RH department and PND on IFA supplementation doses and number of tablets, respectively; reassessment of IFA supplements dose/tablets for prevention vs. treatment of anaemia is needed; community members/women associated supplementation with treatment for anaemia only.	Authors suggested integrating anaemia prevention into family planning (FP) programmes by providing education on maternal nutrition and anaemia prevention during FP counselling, and identifying newly married women and providing IFA supplements.
Nasrat (2014), grey literature, hand search, national, qualitative programme assessment	EPHS/BPHS health facilities' clients	Nutrition component under BPHS and EPHS	Prevention and treatment of malnutrition	None	PND/PPHO understaffed, health staff not trained in nutrition services; most health facilities do not offer complete package of nutrition services, especially at CHCs and BHCs; demand for nutrition services is needed; some providers integrate nutrition education into their other services for mothers; two of 12 health facilities reported stock out of IFA supplements in the 6 months prior to the visit	Nutrition component is under‐staffed and under resourced, optimal nutrition services not delivered through BPHS and EPHS; authors suggest a centralized nutrition supply management to prevent stockout of key nutrition supplies (IFA, vitamin A, micronutrient supplementation)
The World Bank (2013), grey literature, national, qualitative assessment	Household, with focus on women and children 0–23 months	Nutrition and hygiene awareness pilot, part of the Afghanistan safety nets project (unconditional cash transfer), added as a soft conditionality to raise awareness: 2 educational sessions (beginning and end) with a small evaluation in between to improve delivery between the two sessions. Households received food packets and soap cakes; messages on handwashing at key points.	Awareness	Nutrition education with safety net programme	Participants appeared to understand the importance of breastfeeding, but not appropriate complementary feeding; participants misunderstood messages on handwashing before key actions (preparing food or feeding children) compared with after (use of toilet).	No behaviour change data presented; more than two points of contact needed to ensure retention of messages; more focus on tools for targeting husbands and mothers‐in‐law because of their roles as key influencers.
JS Consultancy (2016), grey literature, hand search, subnational, qualitative programme evaluation	Women who had a child 6 months or younger	IEC materials and training for CHWs and FHAG members on individual birth preparedness‐plan cards, maternal nutrition, ANC, delivery care, newborn care, postnatal care, and health education.	Awareness	None	1% and 4% increase in the proportion of women reporting an increase in their food intake during their last pregnancy in the intervention areas of Kandahar and Bamyan, respectively, whereas a decrease was seen in the control areas.	Focus on food consumption increase during pregnancy but not micronutrients, no details on ANC counselling on nutrition
Pedersen, Ayan, Sibghatullah, Erfani, and Noorzad (2016), grey literature, hand search, subnational, qualitative programme evaluation	Children <5 years, mothers	A package of community and facility‐based interventions that provide preventive and curative health and nutrition services, as well as mobilizing communities towards healthy nutrition behaviours and practices.	Package of interventions	Agriculture interventions (home gardens and poultry husbandry for women)	Of the 18 MUNCH interventions, 10 had targets listed in the annual work plans whereas eight did not. Targets were met for latrine building, distribution of micronutrient powders (MNPs), distribution of chickens, and establishing home gardens. Men and women demonstrated improved knowledge regarding the importance of dietary diversification as a method of increasing intake of micronutrients, many also expressed frustration due to the fact that diverse foods were either not available or affordable in their communities.	Many of these interventions are not sustainable without donor support. Home gardens were more productive when access to water was not a challenge; FHAGs were also keys in disseminating information to hard‐to‐reach areas.
GAIN (2017), Grey literature, hand search, national evaluation	Households	National salt iodization, and fortification of wheat flour and oil	Micronutrients	Micronutrient fortification in food	Awareness of fortification was low: 22% of households reported hearing about fortified foods, 35% in Kabul compared with 33% in other urban areas and 20% in rural areas; level of fortification was found to be inconsistent with the national standards: 2% of salt brands, 4% of oil brands, and 10% of wheat flour brands were fortified within the standard range; 71% of salt brands and 51% of wheat flour brands were fortified to some extent, but only 35% of oil brands were fortified at all.	High potential for impact from large‐scale fortification of salt and oil; potential for wheat flour is lower. Further exploration is needed to assess the feasibility of targeting small‐scale producers. For all food vehicles, monitoring, regulation and enforcement are critical for improving the level of fortification, for both domestic and imported products. Future research should assess the nutrient contribution from fortified foods and the total intake of the nutrient from all dietary sources to see if the nutrient gap in the diet is being filled through fortification efforts.
Alim and Hossain (2018), published literature, subnational, RCT, qualitative assessment	Adolescent girls 15–25 years	Combination of nutritional training and collective vegetable gardening as part of an ongoing programme called adolescent Reading Centers	Package of interventions (food production and awareness)	Gardening and vegetable consumption	Increase of 20 percentage points in intervention group's knowledge about vitamin A and its causes of deficiency, anaemia, iron deficiency and its impact on children and pregnant women, amount of food intake during adolescence, cause of night blindness and naming the nutrition sensitive vegetables	Greenhouses in the winter were culturally acceptable for the girls because they aren't seen publicly. Agriculture extension workers should engage adolescent girls to improve knowledge and access to technologies and resources for participation in gardening for consumption.
Siekmans et al. (2018), published literature, subnational, mixed‐methods	Pregnant women	IFA supplementation in BPHS	Micronutrients	None	Barriers to receiving IFA supplementation: Insufficient IFA supplies, perceived insufficient training of CHWs by women, providers reported inadequate knowledge regarding reasons to counsel or prescribe IFA.	Community‐based delivery of IFA and ANC provides earlier and more frequent access.

#### 3.4.1 Nutrition interventions through the health system

Two studies had health education interventions through community‐based health actors, such as community health workers (CHWs) and Family Health Action Group members. The intervention provided information, education, and communications materials and training to CHWs on individual birth preparedness plans, maternal nutrition, ANC, delivery, and PNC. At baseline, more than half of the women reported decreasing food intake or “eating down” during pregnancy to avoid complications. At endline, women in intervention areas who received community‐based health education reportedly increased their food intake during their most recent pregnancy (1% reported increase in food intake in Kandahar and 4% in Bamyan), compared with those in the control areas (JS Consultancy Services and Save the Children, 2016; Ministry of Agriculture Irrigation and Livestock and MoPH [Afghanistan]). Although reported food consumption was measured, food consumption patterns were not linked to message receipt and micronutrient intake changes were not assessed. Although the intervention had several components, other maternal nutrition or ANC components were not reported.

Two assessments were conducted on the BPHS IFA supplementation programme. The 2013 World Bank assessment found that national micronutrient guidelines followed international guidance, but guidance lacked direction for when and how often women should be tested for anaemia and focused on detection and treatment rather than prevention of anaemia. Many inconsistencies were identified between ANC guidelines and PND guidance and conflicting information between dose and number of tablets to be prescribed (World Bank, 2013). Siekmans, Roche, Kung, Desrochers, and De Regil (2018) conducted a multicountry qualitative assessment of IFA supplementation barriers and enablers in 2017 and found that in Afghanistan, health facility staff and CHWs did not receive training on counselling or communication about IFAs and did not have IFA‐specific job aids. Although IFA tablets are included as essential drugs under the BPHS, the number supplied often did not match the recommended dose. One study assessed the national extent of food fortification (household use and market availability), specifically salt iodization and fortification of wheat flour with iron and oil with vitamin A. The study found low awareness of fortification (22% of households) and inconsistent levels of fortification compared with national standards (Global Alliance for Improved Nutrition, 2017). Very low proportions of commercially available brands of staple foods (2% of salt, 4% of oil, and 10% of wheat flour) were fortified within the standard range; 71% of salt brands and 51% of wheat flour brands were fortified to some extent, but only 35% of oil brands was fortified at all.

A 2014 BPHS/EPHS nutrition assessment found that about half of health facility staff were not trained in nutrition services and most assessed health facilities did not provide the full nutrition services package, especially at Comprehensive Health Centers and Basic Health Centers, which are the facilities predominantly providing primary care in Afghanistan (Nasrat, 2014). Every province has one Provincial Nutrition Officer (PNO) responsible for coordinating and monitoring nutrition‐related activities and training BPHS staff in nutrition services. However, the assessment found that due to limited resources, PNOs were unable to provide the required support to facilities, and many health facility staff were unaware of PNOs at the provincial level. Some providers reported integrating nutrition education into other services for mothers ad‐hoc, but it was unclear whether message content and delivery among providers was consistent. This lack of consistency complicates the ability to assess effects. Some providers conducted education sessions in waiting rooms, some conducted cooking demonstrations with nutritious foods, whereas others promoted and distributed conventional information, education, and communications materials. IFA supplement stockouts were also reported by health facilities. Notably, the lack of clarity and standardization of IFA supplement use in the policy was also echoed by participants in this study.

#### 3.4.2 Delivery of a package of household, community, and facility‐based interventions, including multisectoral approaches

One intervention comprised a nutrition and hygiene awareness pilot embedded within the Afghanistan Safety Nets Project, an unconditional cash transfer project. Households also received food packets, soap cakes, and handwashing messages. Authors suggested that participants misunderstood messages about handwashing meant to be done before a task (e.g., preparing food) compared with after specific tasks (e.g., using the toilet), but no quantified behaviour or knowledge change data were presented, and nutrition outcomes were not measured (The World Bank Group, 2014). More than two contacts were recommended to ensure that people retained the messages.

One published article by Alim and Hossain (2018) described a randomized evaluation of an adolescent girls' nutrition education and collective gardening intervention to increase uptake of knowledge and consumption of nutritious foods. They found that greenhouses were effective in the winter and culturally acceptable for girls because they were in a nonpublic space. Authors reported a 20 percentage point increase in the intervention group's knowledge score but the design of the trial was poorly described. Two programme evaluation reports assessed delivery of intervention packages targeting households, communities, and facilities but did not measure nutrition‐related knowledge, attitudes, or practices from beneficiaries. These large‐scale programmes mainly included interventions combining agriculture, livelihoods, education, and health and nutrition components. Grunewald, Hahn, and Rafiq (2008) evaluated 20 projects implemented by the UN Food and Agriculture Organization (FAO) that included integrated livelihoods, nutrition education booklets and posters for students, recipe books for mothers, school and kitchen gardens, and food processing training. Interventions specifically targeting women were kitchen gardens, food processing, and food conservation with marketing training. Overall, they found challenges with the projects' ability to demonstrate change due to lack of consistent indicators across projects at baseline and endline and insufficient data collected. More data on intervention effects on the target groups and models used were needed. Programme implementation challenges included the lack of land ownership and access to resources for women to implement projects with livelihood components; thus, actual ability to achieve diverse food production, processing, and conservation was unclear. Additionally, although nutrition education was included in the overall programme, it was not specifically integrated with women's food production components, resulting in a missed opportunity for combined messaging on nutrition, practical livelihoods, and diverse food consumption. Despite these challenges, authors noted broad reach of the nutrition education programme (estimated 30,000 households) by applying a training of trainers strategy and leveraging existing community groups with trusted leaders, like women's literacy groups, for implementation. Additional positive changes were documented at the policy level through strengthened multisector consultations across ministries by ensuring the inclusion of nutrition objectives in strategies for Ministry of Agriculture Irrigation and Livestock, MoPH, and joint UN programmes. Institutional capacity of the Ministry of Agriculture Irrigation and Livestock to address food and nutrition issues through the establishment of the Home Economics Department was also considered a success.

Pedersen et al. (2016) evaluated a programme package of community and facility‐based interventions including training community WASH groups, providing improved latrines, disseminating messages about dietary diversity, providing training and support to women for home gardens and poultry husbandry, and distributing micronutrient supplements. Of 18 interventions implemented, only 10 had clear targets, and of those, only four were achieved. Participants reported increased knowledge about dietary diversity but expressed frustration at the lack of availability and affordability of such foods in their communities. Neither evaluation was designed to quantify programme impacts, and both were largely qualitative due to the lack of programme data and standard measures.

## 4 DISCUSSION

This scoping review synthesized 20 policies and strategies, 29 data sources and reports, and nine intervention evaluations relevant to improving nutrition in adolescent girls and WRA in Afghanistan. Key findings from this review include the following: (1) There is a triple burden of malnutrition affecting WRA—underweight status, overweight status, and micronutrient deficiencies, especially iron (anaemia) and vitamin D; (2) policies and strategies have transitioned towards multisectoral efforts and have expanded beyond a focus predominantly on infants and children to include nutrition for WRA, specifically targeting pregnant women and adolescent girls; (3) the availability and scope of maternal nutrition data have increased substantially since 2013, yet few nutrition feasibility, process, and efficacy evaluations, nutrition intervention studies and trials, and population surveys include maternal nutrition‐related measures and maternal outcomes; and (4) there is scant evidence about the effectiveness of interventions targeting maternal nutrition and their scale‐up in Afghanistan, a gap that requires urgent attention. Taken together, we believe these findings are adequate to guide programme design and more robust monitoring of nutrition‐sensitive and nutrition‐specific programming for WRA and to be responsive to current policies that are more explicit about nutrition for women.

One example of a reasonable expectation based on our findings is greater standardization and implementation of national programme models that comply with policy. With the strengthened multisectoral support for nutrition programming, the Community Based Nutrition Package (CBNP) has been developed and shaped by policy to target both pregnant women and families with children under 24 months for nutrition, WASH, and food security interventions (MoPH [Afghanistan], 2018). Through this example, we also find greater awareness and planning for evaluations, as the CBNP is being evaluated with a cluster‐randomized trial in three provinces by a collaborative effort between MoPH and key stakeholders. Policy change with multisectoral support and advocacy has been the first step, as shown by evidence in this review. The information presented here suggests that standardized programming may expand at scale, followed by adaptive management with the use of data from evaluations and more rigorous monitoring.

Adolescent girls and pregnant women require additional nutrition for their own health and, ultimately, the health and development of their children. Ensuring access to quality nutrition care for women and adolescent girls will result in intergenerational health and nutrition gains. However, nutrition outcome measures among pregnant women, WRA, and adolescent girls are rarely collected and reported. Few data sources report on indicators that capture women's overall nutrition status, such as BMI and MUAC. Scarcity of nutrition indicator data among women is not limited to Afghanistan—few national surveys, including the DHS, conducted across a range of countries include measures for nutritional status of WRA. Globally, the nutrition data gap is greatest among adolescent girls, because most data, if available, come from small‐scale school‐based surveys (Christian & Smith, 2018). Efforts are needed to strengthen documentation of maternal nutrition status by including maternal nutrition measures in household surveys, monitoring and evaluation of community level nutrition and WASH projects, and routine monitoring through the Health Management and Information System.

Several policies and strategies within the health sector and across development sectors have recognized the need to address maternal malnutrition and associated intergenerational effects and have integrated maternal nutrition strategies into larger reproductive, adolescent, and child health; WASH; and food security strategies. The PND, for example, developed specific policies for addressing micronutrient deficiencies among adolescent girls and WRA and improving IYCF practices. These policies have specifically included content on maternal nutrition and dietary needs such as increasing caloric intake for lactating women, as well as community structures to support and protect women who breastfeed.

The 2013 Maternal and Child Nutrition Lancet Series identified key interventions to rapidly improve nutrition outcomes. Globally, the evidence base has expanded regarding effective nutrition interventions in the preconception period and during pregnancy to improve maternal and fetal outcomes (Vaivada, Gaffey, Das, & Bhutta, 2017). Of the interventions included in our review, four focused on micronutrient supplementation, and the remaining five were related to education about healthy food consumption during pregnancy and hygiene or food‐based programmes involving agriculture and livelihoods. Although micronutrient deficiencies among Afghan women are widespread, nutrition education focuses predominantly on IFA supplementation, with less emphasis on other essential micronutrients for pregnant women and adolescent girls. Similar to other LMICs, in Afghanistan the adolescent period has historically been neglected, yet increased nutrients are essential for adequate growth during adolescence (Christian & Smith, 2018). An increased focus on adolescent girls' nutritional needs may ensure that effective interventions are delivered, such as IFA supplements and increased nutritious food intake through school gardens (Vaivada et al., 2017). Further, protein energy deficiency data are collected in household surveys, but we did not find interventions providing protein energy supplementation or promoting increased protein energy consumption during pregnancy in our search. Additionally, there was no messaging on the dangers of overweight or obesity. Obesity is a growing public health concern in South Asia due to dietary changes, particularly in urban areas, and a consideration of the multifaceted burdens of poor nutrition should be made for populations at risk for obesity. The multisector programmes confirm previous studies revealing the importance of using community‐based channels for implementation, such as volunteer CHWs and Family Health Action Group members (Bhutta et al., 2008; Goudet et al., 2018).

Evidence for effectiveness of large‐scale multisector programmes was scarce because evaluations were designed to assess vertical interventions, not the programme overall. Also, the evaluations used methods that were feasible in the challenging research environment of Afghanistan, which frequently precluded randomized trials. As a result, the data from these studies were excluded from systematic reviews (Goudet et al., 2018). Programme implementation in a fragile context is difficult, and although there are numerous gaps, significant gains and investments across sectors have been made in maternal nutrition in Afghanistan. Despite the challenges, Afghanistan has committed to the Scaling Up Nutrition (SUN) movement, joining 59 countries to reduce malnutrition by 2030 through multisector coordination and bringing line ministries under this common goal. Although many evaluation studies did not measure maternal nutrition‐related outcomes, they documented lessons learned (Table 3) and implementation challenges. Formative research, process evaluations, and implementation research may be more feasible than outcome or impact evaluations and provide much needed evidence for implementation strategies. Further, several cross‐sectional studies have collected data and reported on maternal nutrition‐related measures since the 2013 NNS. A systematic review on barriers and facilitators to improving maternal nutrition in LMICs found that there is scant evidence of programming to support healthy maternal diet and gestational weight gain, including in fragile country contexts. Maternal diet counselling and counselling on weight gain during pregnancy were considered missed opportunities and could be effective if implemented at the community level (Kavle & Landry, 2018). There is limited evidence on effective nutrition programmes to improve maternal nutrition from fragile countries; however, an analysis investigating factors that affect fragile countries from engaging with the SUN initiative found significant associations with their government effectiveness (Taylor et al., 2014). South Asia as a region has a disproportionate burden of global malnutrition, yet neighbouring countries with similar contexts may have experiences and lessons learned that could be applied to Afghanistan. Bangladesh is the only country in the region on track to reducing maternal anaemia to the World Health Assembly target of 40% by 2025, and studies from the region show that a combined community‐based delivery platform combining nutrition supplements and counselling can improve access to and use of supplements by pregnant women (Torlesse & Aguayo, 2018). Other successful intervention examples include the Suahaara‐II project in Nepal, where exposure to community mobilization, mass media promotion, and interpersonal communication and engagement with frontline workers increased maternal dietary diversity in a dose‐response fashion with programme components (Suresh et al., 2019). A meta‐analysis of maternal micronutrient supplement interventions, including studies from Bangladesh, India, Indonesia, Nepal, and Pakistan, found that multiple micronutrient supplements, particularly when started before 20 weeks gestation, were more effective in reducing incidence of preterm birth, low birth weight, and small for gestational age and also reduced maternal mortality among anaemic women (Smith et al., 2017).

This scoping review has limitations for consideration when interpreting the data. We excluded non‐English language documents, potentially excluding relevant information. Although scoping review methodology allows for broad inclusion criteria, we may have missed grey literature that required further searching, particularly for possible nutrition messaging inclusion within ANC and PNC. We endeavoured to keep our inclusion strategy broad, but studies were only eligible if nutrition‐related indicators, including hygiene promotion, were measured among adolescent girls or WRA. We did not conduct a quality review due to limited evidence for efficacy of the evaluated interventions.

The government of Afghanistan, international donors, and implementing partners have made important investments in introducing nutrition‐specific and nutrition‐sensitive interventions to improve maternal nutrition and expand government stewardship for nutrition programming nationally. One example includes the nascent Afghanistan Institute for Nutrition and Home Economics, which, though a private institute, supports different nutrition activities nationally and advocacy and evidence collation for nutrition, including maternal nutrition, nutrition curriculum development, among other objectives. This scoping review presents the evidence spanning more than 10 years of effort to improve the nutrition status of Afghans through policy, data, and programmes. Based on this review, we propose two main recommendations. The first would be indicators on the nutritional status of adolescent girls and WRA should be included in routine household surveys to address serious data deficits, as well as population‐level micronutrient measurements through household surveys. The second would be knowledge and behaviours related to micronutrient deficiencies, supplements, and nutritious and fortified foods must be improved via interventions with documented efficacy and effectiveness. For example, more effective approaches to reach pregnant women are needed to improve the very low IFA supplementation coverage. More thorough engagement is needed to increase knowledge of micronutrient deficiency, the importance of taking supplements during pregnancy, and knowledge about dietary diversity, using multiple communication channels (e.g., mass media, interpersonal counselling, and community campaigns), with appropriate targeting of women, adolescent girls, husbands/fathers, mothers‐in‐laws, and other key influencers. The policy environment is currently adequate to support these recommendations but needs to be complemented by additional domestic and external resources to ensure adequate programming, quality of routine care, and monitoring and evaluation. A combination of these efforts can operationalize the policies and strategies for improved maternal nutrition. The findings of this review may be applied to other fragile settings like Afghanistan to inform strategies and galvanize multisectoral efforts to improve maternal nutrition.

## CONFLICTS OF INTEREST

The authors declare that they have no conflicts of interest.

## CONTRIBUTIONS

CK contributed to study conception and design, collected, extracted, and analysed the data, and drafted the manuscript. GFM contributed to study conception, data extraction and interpretation, and critical review. MOM contributed to data extraction and critical review. PMP, MJA, and MHL contributed to critical review. CST contributed to study conception and design, data extraction, and interpretation and critically revised the draft. All authors read and approved the final version submitted.
